# *piggybac*- and PhiC31-Mediated Genetic Transformation of the Asian Tiger Mosquito, *Aedes albopictus* (Skuse)

**DOI:** 10.1371/journal.pntd.0000788

**Published:** 2010-08-17

**Authors:** Geneviève M. C. Labbé, Derric D. Nimmo, Luke Alphey

**Affiliations:** 1 Oxitec Limited, Oxford, United Kingdom; 2 Division of Biology, Imperial College London Silwood Park, Ascot, United Kingdom; 3 Department of Zoology, University of Oxford, Oxford, United Kingdom; USAMRIID, United States of America

## Abstract

**Background:**

The Asian tiger mosquito, *Aedes albopictus* (Skuse), is a vector of several arboviruses including dengue and chikungunya. This highly invasive species originating from Southeast Asia has travelled the world in the last 30 years and is now established in Europe, North and South America, Africa, the Middle East and the Caribbean. In the absence of vaccine or antiviral drugs, efficient mosquito control strategies are crucial. Conventional control methods have so far failed to control *Ae. albopictus* adequately.

**Methodology/Principal Findings:**

Germline transformation of *Aedes albopictus* was achieved by micro-injection of embryos with a *piggyBac*-based transgene carrying a 3xP3-ECFP marker and an *attP* site, combined with *piggyBac* transposase mRNA and *piggyBac* helper plasmid. Five independent transgenic lines were established, corresponding to an estimated transformation efficiency of 2–3%. Three lines were re-injected with a second-phase plasmid carrying an *attB* site and a 3xP3-DsRed2 marker, combined with PhiC31 integrase mRNA. Successful site-specific integration was observed in all three lines with an estimated transformation efficiency of 2–6%.

**Conclusions/Significance:**

Both *piggybac*- and site-specific PhiC31-mediated germline transformation of *Aedes albopictus* were successfully achieved. This is the first report of *Ae. albopictus* germline transformation and engineering, a key step towards studying and controlling this species using novel molecular techniques and genetic control strategies.

## Author Summary

The Asian tiger mosquito, *Aedes albopictus*, is a highly invasive mosquito and has spread from South East Asia to Europe, the United States and northern areas of Asia in the past 30 years. *Aedes* mosquitoes transmit a range of viral diseases, including dengue and chikungunya. *Aedes albopictus* is generally considered to be somewhat less of a concern in this regard than *Aedes aegypti*. However a recent mutation in the chikungunya virus dramatically increased its transmission by *Aedes albopictus*, causing an important outbreak in the Indian Ocean in 2006 that eventually reached Italy in 2007. This highlights the potential importance of this mosquito, which can thrive much further from the Equator than can *Aedes aegypti*. This paper describes the first genetic engineering of the Asian tiger mosquito. This is an essential step towards the development of genetics-based control methods against this mosquito, and also an invaluable tool for basic research. We describe both transposon-based and site-specific integration methods.

## Introduction

*Aedes* mosquitoes are responsible for an estimated 50 to 100 million dengue cases worldwide every year, with nearly half the world's population at risk of being infected [1], [2]. The two main vector species, *Aedes aegypti* (L.) and *Aedes albopictus* (Skuse) are also the main vectors of the chikungunya virus, which can cause severely debilitating syndromes lasting up to several months.

In the last 30 years, *Ae. albopictus* has travelled the world via human travel and commerce, e.g. the trade of used tyres [3] and “lucky bamboo” [4]. It spread from Southeast Asia and Pacific Islands to Europe, North and South America, Africa, the Middle East and the Caribbean. Its strong ecological plasticity, including the ability of some strains to lay diapausing eggs that can survive cold winters, makes it a highly invasive species [5], [6]. It is now established as far north as Switzerland in Europe [7] and Illinois in the USA [8], where winter conditions preclude the establishment of *Ae. aegypti* [9].

The introduction of *Ae. albopictus* in the USA was once perceived as a net benefit for public health as it often displaced the more competent vector of dengue, *Ae. aegypti* [10], [11], [12]. However, a single nucleotide mutation of the chikungunya virus appears to have considerably enhanced its infectivity in *Ae. albopictus* [13], [14] leading to chikungunya outbreaks in the Indian Ocean in 2006–2007 [15], including an epidemic affecting a third of the population (260,000 cases) in La Réunion Island [16] where *Ae. albopictus* is the sole vector. Transmission also occurred in Italy in 2007 [17], confirming that *Ae. albopictus* is indeed a route for tropical diseases to extend their geographic range into more temperate countries.

In the absence of vaccine or antiviral drugs for either chikungunya or dengue, efficient mosquito control strategies are crucial. Conventional control methods (insecticide spraying and management of breeding sites) have so far failed adequately to control *Ae. albopictus*. The 2007 ECDC report on risk assessment of chikungunya in EU states that “once *Aedes albopictus* is known to be established in an area, it is difficult (not to say impossible) to eradicate the mosquito” [1].

Novel control methods are being developed that involve the use of genetically modified mosquitoes to either suppress the target population or replace it with a pathogen-resistant strain [18], [19], [20], [21]. Transgenesis is an essential tool required to develop these genetics-based control methods. It is therefore highly desirable to establish germline transformation of *Ae. albopictus*. Germline transformation of a number of insect species, including *Aedes aegypti* mosquitoes, is now routine through the use of transposable elements. The most commonly used is *piggyBac*, a class II transposable element that inserts into TTAA sequences [22]. *piggyBac* has been used successfully to transform a wide range of insect species from several orders, including Diptera, Lepidoptera, Hymenoptera and Coleoptera (reviewed in [23]). Among insects, transformation efficiency using *piggyBac* is typically 3–13%, and 4–11% in *Aedes aegypti* in particular [24], [25], [26], [27], [28]. The short recognition sequence of transposable elements leads to effectively random integrations into the host genome [29].

Positional effects and possible gene disruptions affect both transgene expression and fitness of the transgenic lines, so that a single transgene may lead to a range of phenotypes depending on its insertion site. In some cases it may be useful to have several lines with slightly different phenotypes to choose from; however this random integration pattern makes it difficult to compare two different transgenes as their different phenotypes are an unknown combination of the inherent properties of the transgene and the effects of the insertion sites.

Site-specific transgene integration systems have been developed using recombination systems which target a specific nucleotide sequence that is long enough that it is unlikely to occur naturally in an insect genome. Examples include Cre-*loxP* from bacteriophage P1 [30], Flp-*FRT* from the 2 micron plasmid of *Saccharomyces cerevisiae* [31] and phiC31-*att* from a *Streptomyces* bacteriophage [32]. In each case one of the target sequences for the recombinase is introduced as a “docking site” into the genome of interest using a transposable element-based “first phase” transgene. Second-phase transgenes can then be inserted repeatedly into the preferred docking site using the appropriate recombination enzyme [28]. Although those integration systems have all been demonstrated in *Drosophila* [33], [34], only the phiC31-*att* system has been used successfully to integrate a transgene in *Ae. aegypti* [28]. The phiC31 integrase catalyses a unidirectional recombination between so-called *attB* and *attP* sites, creating *attL* and *attR* junctions [35]. Typically, *attP* is used as the docking site for *attB*-carrying transgenes. In *Ae. aegypti*, the transformation efficiency using the phiC31-*att* system was reported to be 17–32% [28].

Here we report the use of a *piggyBac*-based system to achieve the first successful germline transformation of *Aedes albopictus*. Further, we describe successful site-specific integration into the *Ae. albopictus* genome using the PhiC31-*att* system.

## Materials and Methods

### Plasmid constructs

**Figure 1:**
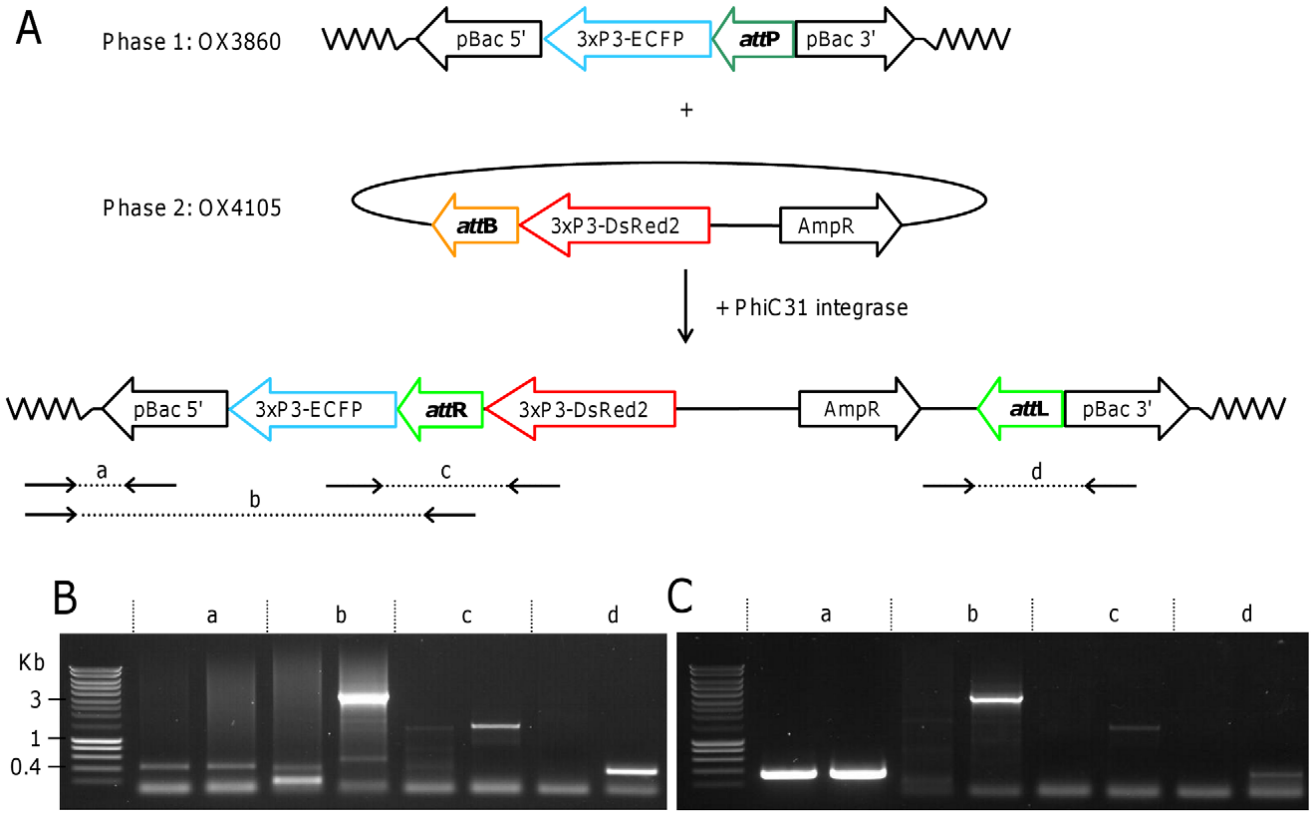
PhiC31-mediated site-specific integration of the OX4105 into OX3860 lines of *Aedes albopictus*. A: The *attP* docking site was inserted into the *Aedes albopictus* genome using the *piggyBac*-based vector OX3860. The OX4105 construct containing an *attB* site was then injected together with mRNA encoding PhiC31 integrase. The expected structure following site-specific integration is represented. The structure of actual insertions was analysed by PCR amplification using primer pairs ‘a’ (3860B-5′flank1 or 3860C-5′flank1 with PB2; 426bp or 363bp, respectively), ‘b’ (3860B-5′flank1 or 3860C-5′flank1 with Diag-attBD, approx. 3kb), ‘c’ (Diag2-ECFP with Diag-DsRed2, 1208bp) and ‘d’ (pBac-3′R with M13-28-R, 372bp). B, C: PCR amplifications using primer pairs ‘a’, ‘b’, ‘c’ and ‘d’ on gDNA from lines OX3860B and OX3860C, respectively. For each primer pair, the left and right lanes correspond, respectively, to gDNA before and after the insertion of the OX4105 construct. In each case the band sizes after insertion correspond to those expected from canonical insertion events as illustrated in panel A. Representative bands were sequenced; these data confirmed that the insertions had the expected structure (data not shown). Equivalent results were obtained for insertion of the OX4105 construct into line OX3860A. The size marker is Smartladder (Eurogentech, Southampton, UK).

The OX3860 construct is the pBac[3xP3-ECFPaf]-attP plasmid described by Nimmo *et al.* [28]. The construct OX4105 carries an *attB* site and a 3xP3-DsRed2 marker, and was designed to integrate into the OX3860 construct such that, after integration, the two markers would be in the same orientation (Figure 1). This allows the comparison of the expression of the two markers in an equivalent genomic context.

The OX4105 construct was made by modifying pBattB[3xP3-DsRed2nls-SV40]lox66 [28] to remove the nuclear localisation signal of the DsRed2 protein and change the orientation of the *attB* sequence. The DsRed2-nls-SV40-lox66 cassette was removed using *Age*I/*Not*I and replaced with an *Age*I-*Eag*I DsRed2-SV40 cassette to create pBattB[3xP3-DsRed2-SV40]. The original *attB* cassette was removed from pBattB[3xP3-DsRed2-SV40] using *Xho*I, creating pB[3xP3-DsRed2-SV40]. The *Kpn*I/*Sac*II *attB* fragment from pBattB[3xP3-DsRed2-SV40] was subcloned from pBattB[3xP3-DsRed2-SV40] into pSLfa1180fa and the *Sac*II/*EcoR*V fragment from this plasmid was then cloned into the *Sac*II/*Swa*I sites of pB[3xP3-DsRed2-SV40] creating OX4105.

### Insect strains and rearing

The *Ae. albopictus* wild-type strain was colonised in 2006 from Malaysia (Institute of Medical Research, Kuala Lumpur). The strain was reared at 27°C (±1°C) and 80% (±10%) relative humidity. Larvae were fed on crushed dry fish food (TetraMin® flake food from Tetra GmbH, Germany) and adults on 10% glucose with 14U/ml penicillin and 14 µg/ml streptomycin. Females were fed on horse blood using a *Hemotek* Insect Feeding System (Discovery Workshops, Accrington, UK) set at 37°C.

### Microinjection of *Aedes albopictus*

Pre-blastoderm embryos were injected as described by Morris *et al.* [36] except that cover slips of injected embryos were placed vertically into water in order to drain the oil for at least an hour, and then immediately placed vertically in a sealed humid box for 4 days. To produce donor *att*P strains, wild-type embryos were injected with a mixture of the OX3860 construct (300 ng/µl), phsp-Bac plasmid helper (200 ng/µl) [37] and *piggyBac* mRNA (300 ng/µl) in injection buffer (5mM KCl and 0.1 mM NaH_2_PO_4_, pH 6.8). Though, in principle, either mRNA helper or helper plasmid should be capable of mediating transformation, we co-injected both together to provide a degree of redundancy and in order to increase chances of successful transformation. The *piggyBac* mRNA was transcribed from OX3081 construct (*piggyBac* transposase coding sequence under the control of the T7 promoter [38] and the 3′UTR from the *DmVasa* gene [39]) using the mMESSAGE mMACHINE® T7 kit (Ambion, Austin, TX). The mRNA was purified using the MEGAclear™ kit (Ambion), precipitated with ammonium acetate and resuspended in 10 µl nuclease-free water. For site-specific integration, embryos from the donor strains were injected with OX4105 (350 ng/µl) and PhiC31 mRNA (600 ng/µl) [28] in injection buffer. The PhiC31 mRNA was transcribed and purified using the mMESSAGE mMACHINE® T7 and MEGAclear™ kit (Ambion). Construct and helper plasmids were purified using the EndoFree Plasmid Maxi kit (Qiagen, Hilden, Germany). Larvae were screened for fluorescence using a Leica MZ95 microscope with the appropriate filter sets from Chroma Technology (Rockingham, VT) (filters: ECFP: exciter D436/20x; emitter D480/40m; DsRed2: exciter HQ545/30x; emitter HQ620/60m). Pictures of fluorescent larvae were taken with Canon PowerShot S5IS with an MM99 adaptor (Martin microscopes) to fit into the eyepiece.

### Inverse PCR

Inverse PCR was performed essentially as described by Handler *et al.* [40]. Genomic DNA from each line was extracted using the NucleoSpin Tissue kit (Macherey-Nagel). 2.5 µg of gDNA was cut with the restriction enzymes *Hae*III, *Msp*I, *Taq*I and *Dpn*II. PCR was performed using 2 µl of digested genomic DNA, *Taq* DNA polymerase with Thermopol buffer (New England BioLabs, Ipswitch, MA) and either the *piggyBac* 5′ or 3′ primer pair (5′ forward: tcttgaccttgccacagagg; 5′ reverse: tgacacttaccgcattgaca; 3′ forward: gtcagtccagaaacaactttggc; 3′ reverse: cctcgatatacagaccgataaaaacacatg). The thermal cycling parameters were 95°C for 5min, followed by 35 cycles of (95°C for 30sec, 55°C for 1min, and 68°C for 2 min), and a final extension step of 72°C for 10 min.

PCR fragments were extracted using the Minelute Gel Extraction kit (Qiagen), cloned into pJet vectors (GeneJET PCR cloning kit from Fermentas, Vilnius, Lithuania) and transformed into XL-10 cells (Stratagene, La Jolla, CA). Positive clones were purified (GeneJET Plasmid Miniprep Kit, Fermentas) and sent for sequencing (GATC Biotech, Germany).

### PCR analysis of site-specific integration

Integration of OX4105 into donor strains was investigated using primers in the 5′ flanking genomic sequence (3860C-5′flank1: cacaatggaaccatgaaaacttaaaccag; 3860B-5′flank1: tgagaacaagatggcgattctaggagt) with a primer in the attR sequence (Diag-attBD: tgatggaccagatgggtgagg) or in the 5′ *piggyBac* end (PB2: cagtgacacttaccgcattgacaag).

The attR junctions were amplified and sequenced using primers in DsRed2 (Diag-DsRed2: ctgggaggcctccaccgagc or Diag3-Dsred: cacctcccacaacgaggactac) and ECFP (Diag2-ECFP: acagctcctcgcccttgctca). The attL junctions were amplified and sequenced using primers in the 3′ *piggyBac* fragment (pBac3′R: tggaccttttctcccttgctactgac; Diag-pb3: ttccgtacaataatgccataggccac) and in the OX4105 backbone (M13-28-R: tgtgagcggataacaatttcacacagga; M13-RP (GATC Biotech): caggaaacagctatgacc). PCR fragments were purified using the Minelute Gel Extraction kit (Qiagen) and sent for sequencing to GATC Biotech.

## Results

### Germline transformation of *Aedes albopictus* with the *piggyBac* transposable element

Five independent transgenic lines were established using the *attP*-containing *piggyBac* construct OX3860 (transgenic lines OX3860A, B, C, D and F). Approximately 6000 eggs were injected and approximately 1500 larvae hatched. This corresponds to 25% survival post-injection, which is comparable to the 20.5% and 23.3% survival obtained in *Aedes aegypti* by Kokoza *et al.* [24] and Nimmo *et al.* [28], respectively. In total, approximately 250 males survived to be crossed with wild-type females in pools of four males for 24 hours before being merged into seven pools, and 300 females were crossed with wild-type males in three pools. In preliminary experiments this seemed to give more reliable production of G_2_, though there was also a risk that the G_2_ embryos from such pools may not represent all fertile G_0_ parents. The five transgenic lines originated from two G_0_ male pools and two G_0_ female pools. The B and C lines originated from the same male G_0_ pool but were easily distinguished by fluorescence phenotype. Mendelian inheritance data are provided in Table S1.

Inverse PCR analysis based on their different fluorescent phenotypes of insertions showed that all the lines were independent insertions and showed the typical targeting and duplication of a TTAA sequence by the *piggyBac* element (Table 1). These sequences could not be used to directly locate the insertions, as the genome of *Ae. albopictus* has not been sequenced. Inverse PCR results failed to identify a second insertion in the OX3860C line, which was discovered after second-phase insertion (see PhiC31 intergration results below).

**Table 1:** Flanking sequences of integration sites of OX3860 into *Aedes albopictus*.

Strain	5′ Flanking sequence		3′ Flanking sequence
OX3860A	n.d.	ttaa	tcaactcaacgtacatatgta
OX3860B	gcgcacaagcttagaggtact	ttaa	tccaagcagacaaccgaaatg
OX3860C	cctgacgtgactagataaccc	ttaa	ggaatgagtaactcttggtag
OX3860D	tttactaacacaaaattagta	ttaa	cgtcattcgttttgcagaaga
OX3860F	cttccatgtagattgtttcgt	ttaa	acgtccgtgaaatagtatcgc

Genomic sequences immediately flanking the *piggyBac* insertions of OX3860 lines were obtained by inverse PCR. All the insertion sites were unique and occurred at a TTAA site, the canonical recognition sequence for the *piggyBac* transposable element. n.d.: not determined. The 5′ inverse PCR for the OX3860A line was not successful but the 3′ flanking sequence is sufficient to prove the independence of the A insertion. Full flanking sequences are provided in Table S2.

The 3xP3-ECFP marker [41] showed the expected fluorescence in the larval eyes in lines OX3860B, C, D and F. Line OX3860B also showed strong expression in the anal papillae of larvae, which has been previously observed in *Aedes aegypti* [28]. Line OX3860A exhibited an unusual expression pattern, with variable fluorescence intensity between individuals, and between the two eyes of an individual, but PCR analysis confirmed that they all carried the same integration event (data not shown). In addition, progeny from larvae showing weak fluorescence in one eye included individuals with intense fluorescence in both eyes. The variation in fluorescence observed in individuals from the OX3860A line is likely due to unusually strong position effects of adjacent genomic elements, or position effect variegation [42].

The OX3860F insertion was linked to the male-determining locus, the two loci being approximately 8.5 centiMorgans (cM) apart (Table 2).

**Table 2:** The OX3860F insertion is linked to the male-determining locus.

Males:	322 (52%)	Fluorescent:	298 (93%)
		Wild Type:	24 (7%)
Females:	302 (48%)	Fluorescent:	29 (10%)
		Wild Type:	273 (90%)

The progeny from a cross between heterozygous OX3860F males (G_6_) and wild-type females shows that transgene transmission is highly skewed towards male progeny (93% of male progeny expressed the marker, versus only 10% of the female progeny, n = 624). The sex-ratio, however, is normal, indicating a male-linked insertion rather than female lethality. Non-parental phenotype was observed in 8.5% of the progeny, indicating a distance of 8.5 centiMorgans (cM) between the insertion and the male-determining locus.

Transformation efficiency is usually defined as the proportion of fertile (G_0_) injection survivors giving at least one transformed (G_1_) progeny. In preliminary studies, difficulties had been encountered in getting females to feed and lay when kept individually, so G_0_ females were pooled; it is therefore not possible to determine the fertility rate post-injection or to calculate precisely the transformation efficiency. The transformation efficiency was at least 1% in these experiments (six independent insertions from 550 G_0_ adults) Table 3. If we assume that the fertility rate of G_0_ adults is similar to *Aedes aegypti* then we can estimate that the transformation efficiency was 2–3%. For comparison, the range of efficiency of *Ae. aegypti* transformation is between 4–11% [24], [25], [28].

**Table 3:** Transformation efficiency of *Aedes albopictus* with *piggyBac* (OX3860) and *attB* (OX4105) constructs.

Strain	Construct	Eggs injected	Survival to pupae	G_0_ Adults	Integration events	Transformation efficiency (assuming 30–50% fertile adults)
WT	OX3860	∼6000	1272 (∼21%)	550	6	2.2–3.6%
OX3860A	OX4105	604	36 (6%)	32	≥1	6.2–10.4%
OX3860B	OX4105	2052	303 (15%)	86	1	2.3–3.9%
OX3860C	OX4105	2165	477 (22%)	161	≥2	2.5–4.1%

The “phase 1” *piggyBac* OX3860 construct, carrying an *attP* docking-site, was injected into a wild-type background together with *piggyBac* transposase mRNA. Five lines (OX3860A, B, C, D, F) were obtained including one with two integration events (OX3860C). Three of the resulting OX3860 lines were injected with the “phase 2” OX4105 construct, carrying an *attB* site, together with PhiC31 integrase mRNA. All the wild-type G_0_ pupae were discarded since they did not carry an *attP* site. The OX3860B and C lines were kept heterozygous and about half of the G_0_ pupae were wild-type. The OX3860A line was enriched and all the G_0_ pupae out of those injections were transgenic: a mixture of heterozygote and homozygote individuals. This difference could explain the higher transformation efficiency observed in the OX3860A background.

### PhiC31-mediated site-specific integration

The OX3860 lines carry a PhiC31 *attP* site and therefore allowed us to test the PhiC31 integration system. The OX4105 construct carries a 3xP3-DsRed2 marker and an *attB* site to integrate into attP (Figure 1). Embryos from the OX3860A, B and C lines were injected with OX4105 and PhiC31 integrase mRNA. Survivors were mated to wild-type and their progeny was screened for fluorescence. Successful integration was identified as insects expressing DsRed2 in the eyes in addition to the ECFP (cyan) fluorescence. For lines OX3860B and OX3860C, the injected eggs were derived from a backcross of OX3860[B or C] with wild type and therefore comprised a mixture of heterozygotes (with one copy of the *attP* site) and wild-type. For line OX3860A, the injected eggs were derived from a more inbred line and therefore also contained homozygotes. Wild type injection survivors (G_0_) – lacking the 3xP3-ECFP marker from the OX3860 construct - were discarded as they lacked an *att*P docking site.

For the OX3860A line, 604 eggs were injected, out of which 36 survived to pupae (6%), all of which expressed the cyan fluorescence in the eyes and therefore carried at least one copy of OX3860A. Twelve G_0_ males were crossed in one cage with wild-type females and 20 G_0_ females in a cage with wild-type males. The male cage produced 72 transgenic larvae out of approximately 1100 larvae screened. The G_0_ females gave no transgenic progeny. This corresponds to a minimum transformation efficiency of 3.125% if all of the G_0_ were fertile, 6.25% if half were sterile. This assumes that the 72 transgenic larvae are all derived from a single transformation event and G_0_ parent. This may well be an underestimate, however, since all the integration events occur into the same docking site, independent events within one pool cannot be distinguished.

For the OX3860B line, 2052 eggs were injected, of which 303 survived to pupa (15%). Of these, 154 were wild-type and discarded, since they did not carry an *attP* site. Thirty-six G_0_ males were allowed to mate with wild-type females in three pools of 12 males each, and 50 G_0_ females were crossed together with wild-type males in a single pool. One male cross produced one male and three female transgenic offspring which were reared separately. Only one of the transgenic females gave progeny, starting the line OX4105[3860B]. The minimum calculated integration efficiency is 1.16%, although assuming 50% sterility the estimated efficiency is 2.32%.

For OX3860C, 2165 eggs were injected, out of which 477 survived to pupa (22.0%). Of these, 244 were wild-type and discarded. G_0_ adults were crossed in pools: 71 G_0_ males were crossed in cages of 23, 36 and 12 males with wild-type females, and 90 G_0_ females were crossed in cages of 50 and 40 females with wild-type males. High numbers (>20) of second-phase transgenic G_1_ individuals were found from the first two male G_0_ cages. Within the fluorescent G_1_ individuals, two second-phase fluorescence patterns were observed: individuals with bright red eyes and bright blue eyes (found in the progeny of one G_0_ pool only, named line 1), and individuals with bright red eyes and weakly fluorescent blue eyes (found in the progeny from both positive male G_0_ cages, named lines 2 and 3). Analysis of line 1's progeny (G_2_) showed 198 larvae with bright blue and bright red eyes, 220 wild-type larvae, eight with blue eyes only (named line OX3860C1) and 11 with bright red and weakly blue eyes (named line 4). PCR analysis of genomic DNA from line 1 showed positive amplification of both an empty *attP* site from the OX3860 construct and the *attL* and *attR* junctions characteristic of a site-specific integration into *attP*. Those results led to the conclusion that the OX3860C parent from line 1 had two linked *attP* sites: one that integrated the OX4105 construct and one that stayed free. The linked sites separated in some of the G_2_ individuals, giving OX3860C1 (with blue eyes only, *attP* site without integration) and line 4 (red eyes due to the insertion of the OX4105 construct, and weaker blue eyes due to the loss of the C1 insertion). Nineteen progeny with a non-parental phenotype out of 437 indicates a distance of 4.35cM between the two *attP* sites. Further PCR analysis showed that lines 1, 2, and 3 inserted the OX4105 construct into the same one *attP* site for which the flanking sequence was originally found, and are therefore equivalent insertions (data not shown). Lines 1 and 3 come from the same G_0_ pool and have the same site-specific integration event: they may come from the same parent. We therefore have evidence of only two independent events, giving a minimum estimated transformation efficiency of 1.24% if all the G_0_ were fertile.

**Figure 2:**
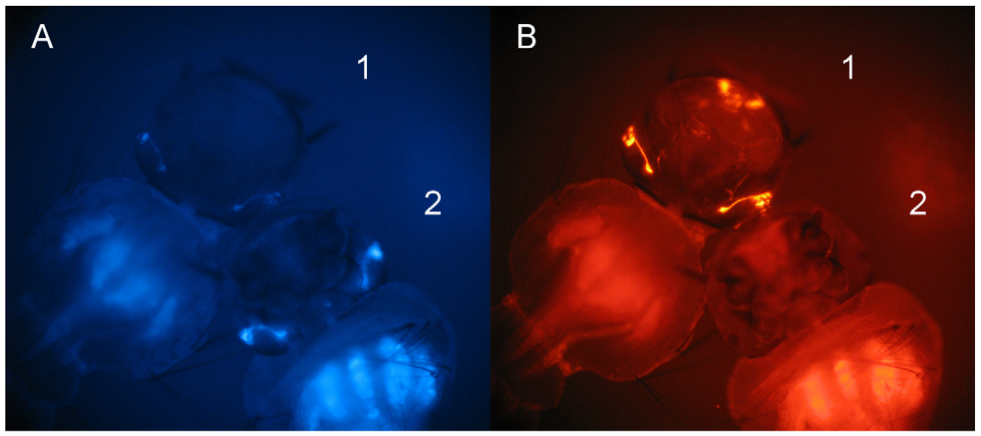
Phenotype of transgenic *Aedes albopictus* OX3860B larvae, with and without PhiC31-mediated site-specific integration of the OX4105 construct. Fluorescence micrographs of two transgenic larvae are shown illustrating the cyan (A) and red (B) fluorescence profiles of each genotype. Larva 1 is OX3860B; larva 2 is OX4105[3860B]. OX3860B larvae carry only the 3xP3-ECFP marker. OX4105[3860B] individuals have both 3xP3-ECFP and 3xP3-DsRed2, giving cyan and red fluorescent eyes. The integration of the OX4105 construct into OX3860 lines appears to reduce the expression of the OX3860 marker (panel A, compare cyan expression of these larvae, each carrying the same 3xP3-ECFP marker). This effect was seen with OX4105 integrations into each of the OX3680 docking sites (data not shown).

In all OX3860 lines, the site-specific integration of 3xP3-DsRed2 showed the same expression pattern as the 3xP3-ECFP of the parental line: variable intensities in OX4105[3860A] individuals, strong anal papillae expression in OX4105[3860B] larvae, eye-only expression in OX4105[3860C] individuals. This is consistent with the two markers being exposed to the same positional effects and was also observed in site-specific integration in *Ae. aegypti* [28]. However the integration of the OX4105 construct appears to have weakened the expression of the 3xP3-ECFP marker in all the lines (Figure 2). This may perhaps be due to a titration of the transcription factors by the addition of a second 3xP3 promoter nearby, or to mechanical interference such as promoter occlusion [43] or dislodgement of a translation-initiation complex by a RNA polymerase transcribing from an upstream promoter [44]. Mechanical interference would imply imperfect function of the SV40 terminator, which has previously been observed in insect cells [45].

PCR characterisation of all three OX4105[3860] lines (Figure 1) confirmed the insertion of the OX4105 construct into the *attP* sites from the OX3860 construct, with a canonical *attP-attB* recombination verified by sequencing of the *attR* and *attL* PCR fragments.

## Discussion

This paper presents the first germline transformation of *Aedes albopictus*. This used a *piggyBac* transposable element, with an estimated transformation efficiency of 2–3%, assuming a post-injection fertility rate similar to *Ae. aegypti* [28].

*Ae. albopictus* has proved particularly difficult to suppress using conventional control methods and hopes reside in new technologies. The development of genetically modified strains has the potential to improve the efficiency of the Sterile Insect Technique (SIT), a method developed in the 1950s that aims at suppressing pest insect populations by releasing males sterilised by irradiation, thereby reducing the proportion of fertile matings in the wild [46], [47]. Transgenic strains can be engineered to carry a genetic marker or a sexing system that would help monitoring the program in the field or automatically eliminating females before releasing the males [48], [49]. The RIDL® system (Release of Insects carrying a Dominant Lethal) [50] – a variation of the Sterile Insect Technique which replaces irradiation by genetically engineered sterility – could also be envisaged [51], . RIDL seems especially interesting for mosquito control since irradiation is particularly damaging to male mosquitoes [54]. Population replacement is another proposed control strategy that could be envisaged, involving genetically engineered strains resistant to the dengue virus [55]. Population suppression, however, would have the advantage of controlling both dengue and chikungunya, while reducing the biting nuisance at the same time.

Genetic vectors based on transposable elements have the potential to remobilise if exposed to a transposase source. However, the efficiency of such reactions seems to vary significantly from one species to another. In fruit flies, the high susceptibility of *piggyBac* to remobilisation allows the removal of *piggyBac* ends from transgenic lines, leaving a stable vector in the genome [56], [57]. Attempts to remobilise *piggyBac* in *Aedes aegypti* by artificial exposure to transposase were unsuccessful [58], suggesting that it may be an ideal choice of vector for the production of transgenic *Aedes* for release programmes.

Site-specific integration is an interesting tool allowing direct comparison of two or more transgenes in a particular genomic environment. Out of the five lines produced using the OX3860 construct, three different fluorescence patterns were observed, highlighting the importance of position effects in transgene regulation. Three different lines were successfully transformed by site-specific integration using the PhiC31 integrase and a donor plasmid carrying an *attB* site. The expression profile of the “phase 2” (site-specifically inserted) marker is similar to that of the corresponding “phase 1” marker, indicating that those two elements, separated by 1922 bp, are subjected to similar influence from the surrounding genomic elements. Site-specific integration occurred successfully in only one of the two available *attP* sites from the OX3860C line, albeit with only a small number of independent events detected. This may indicate that the genomic position of the docking site affect the efficiency of PhiC31-mediated integration. The estimated transformation efficiency with PhiC31 was between 2.3 and 6.3%, depending on the lines. The higher transformation efficiency observed in the OX3860A background is possibly due to the presence of homozygous OX3860A individuals among the population of injected eggs; these have two copies of the *attP* site which may lead to a higher integration frequency for the OX4105 transgene.

*Aedes albopictus* is quickly growing into a major public health threat throughout the world and consequently the subject of numerous research programs. Genetic transformation and engineering is a key step towards studying and controlling this species using novel molecular techniques and genetic control strategies. Investigations would also greatly benefit from a genome sequencing project.

## Supporting Information

Alternative Language Abstract S1 (0.03 MB DOC)

Alternative Language Abstract S2 (0.03 MB DOC)

Table S1 Mendelian inheritance of the transgene in OX3860 lines.
(0.03 MB DOC)

Table S2 Full flanking sequences of interation sites of OX3860 into *Aedes albopictus*.
(0.03 MB DOC)
