# Supplementary material for: piggybac- and PhiC31-Mediated Genetic Transformation of the Asian Tiger Mosquito, Aedes albopictus (Skuse)
Source: PLoS Negl Trop Dis. 2010 Aug 17;4(8):e788. doi: 10.1371/journal.pntd.0000788 (PMC2923142; doi:10.1371/journal.pntd.0000788)
Supplement: Alternative Language Abstract S1 — (0.03 MB DOC) [file pntd.0000788.s001.doc]

**Résumé en français**

**Contexte**

Le moustique tigre *Aedes albopictus* (Skuse) est un vecteur de plusieurs arbovirus, dont celui de la dengue et du chikungunya. Cette espèce originaire d’Asie du Sud-Est est particulièrement invasive et s’est répandue à travers le monde depuis les trente dernières années et est désormais établie en Europe, Amérique du Nord et du Sud, Afrique, au Moyen Orient et dans les Caraïbes. Les méthodes conventionnelles de démoustication n’ont jusqu'à présent pas suffit à contrôler l’expansion d’*Ae. albopictus*. En l’absence de vaccins et de médicaments antiviraux, il est donc crucial de développer de nouvelles méthodes efficaces de lutte anti-vectorielle.

**Méthodologie / Principaux résultats**

La transformation germinale d’*Aedes albopictus* a été accomplie par micro-injection embryonnaire d’un transgène de type *piggyBac* portant le marqueur 3xP3-ECFP et un site *attP*. La transposase *piggyBac* a été co-injectée sous forme d’ARNm et d’un vecteur helper. Cinq lignées indépendantes ont été établies, avec une efficacité de transformation estimée à 2-3%. Trois de ces lignées ont été réinjectées avec un plasmide de deuxième phase portant un site *attB* et un marqueur 3xP3-DsRed2. L’intégrase PhiC31 a été co-injectée sous forme d’ARNm. Dans les trois lignées, le second plasmide a été intégré spécifiquement dans le site *attP*, avec une efficacité estimée à 2-6%.

**Conclusion**

La transformation germinale d’*Ae. albopictus* a été accomplie par deux différentes méthodes : a) un transgène dérivé du transposon *piggyBac*; b) de façon spécifique de site par recombinaison *attP*/*attB* grâce à l’activité catalytique de l’intégrase PhiC31. Ceci constitue le premier rapport de transformation germinale et de modification génétique d’*Ae. albopictus*, une étape fondamentale vers l’application de méthodes innovantes de biologie moléculaire et de contrôle vectoriel à cette espèce.
