# Supplementary material for: piggybac- and PhiC31-Mediated Genetic Transformation of the Asian Tiger Mosquito, Aedes albopictus (Skuse)
Source: PLoS Negl Trop Dis. 2010 Aug 17;4(8):e788. doi: 10.1371/journal.pntd.0000788 (PMC2923142; doi:10.1371/journal.pntd.0000788)
Supplement: Alternative Language Abstract S2 — (0.03 MB DOC) [file pntd.0000788.s002.doc]

**Resumen en Español**

**Contexto**

El mosquito-tigre asiático *Aedes albopictus* (Skuse) es vector de múltiples arbovirus, incluyendo los agentes causales del dengue y de la fiebre chikungunya. Esta especie de mosquito, originaria del sudeste asiático, es particularmente invasiva y se ha diseminado a través del mundo en los últimos treinta años, logrando establecerse en Europa, America del Norte y del Sur, África, el Oriente Medio y el Caribe. Hasta el momento, las herramientas tradicionales de lucha anti-vectorial no han sido suficientes para controlar la expansión de *Ae. albopictus*. Debido a la ausencia de vacunas y medicamentos antivirales, es crucial desarrollar métodos eficaces para el control de poblaciones de mosquitos.

**Metodología / Principales Resultados**

La transformación de la línea germinal de *Ae. albopictus* se realizo mediante micro-inyección embrionaria del transgen asociado con una versión modificada del transposón *piggyBac* que contiene el marcador 3xP3-ECFP y un sitio de integración *attP*. Este vector fue inyectado en combinación con ARNm correspondiente a la enzima *piggyBac*-transposasa y un plasmido ‘ayudante’ que contiene el gen de la misma enzima. Se establecieron cinco linajes independientes, con una eficiencia de transformación del 2-3%. Tres de estos linajes fueron re-inyectados con un plásmido de segunda fase que contiene un sitio *attB* y el marcador 3xP3-DsRed2. Este plásmido fue inyectado en combinación con ARNm correspondiente a la integrasa PhiC31. En estas tres líneas, el segundo plásmido se integró específicamente en el sitio *attP*, con una eficiencia estimada del 2-6%.

**Conclusión**

La transformación germinal de *Ae. albopictus* se completó satisfactoriamente mediante el uso de dos tecnologías: a) un vector basado en el transposón *piggyBac*; b) el sistema *attP*/*attB* catalizado por la integrasa PhiC31, el cual permitió la integración del transgen en un sitio especifico del genoma durante la segunda fase del proyecto. Este es el primer reporte de transformación germinal y de modificación genética en *Ae. albopictus,* y representa un paso fundamental para el desarrollo de métodos innovadores basados en técnicas moleculares y de control genético en esta especie.
