# Supplementary material for: piggybac- and PhiC31-Mediated Genetic Transformation of the Asian Tiger Mosquito, Aedes albopictus (Skuse)
Source: PLoS Negl Trop Dis. 2010 Aug 17;4(8):e788. doi: 10.1371/journal.pntd.0000788 (PMC2923142; doi:10.1371/journal.pntd.0000788)
Supplement: Table S1 — Mendelian inheritance of the transgene in OX3860 lines. (0.03 MB DOC) [file pntd.0000788.s003.doc]

**Supporting Table S**1. Mendelian inheritance of the transgene in OX3860 lines.

| **Line** | **OX3860A** | **OX3860B** | **OX3860C** | **OX3860D** | **OX3860F** |
| --- | --- | --- | --- | --- | --- |
| WT | 264 | 664 | 976 | 7 | 297 |
| Transgenic | 288 | 583 | 943 | 10 | 327 |
| P value (χ^2^) | 0.307 | 0.0218 | 0.4513 | 0.4669 | 0.2298 |

For each insertion line except OX36860B, the progeny of heterozygotes crossed to wild-type showed Mendelian inheritance of the transgene (not significantly different from 50:50 transgenic versus wild-type ratio). This is consistent with each line carrying a single insertion. For line B, the lower proportion of transgenic progeny may indicate a fitness cost associated with the transgene. Those observations were made on G_2_ (line D, hence the small numbers), G_3_ (lines A, B, C) or G_6_ (line F) generations. Note that other data subsequently showed that OX3860C in fact carries two closely linked transgene insertions.
