# Supplementary material for: piggybac- and PhiC31-Mediated Genetic Transformation of the Asian Tiger Mosquito, Aedes albopictus (Skuse)
Source: PLoS Negl Trop Dis. 2010 Aug 17;4(8):e788. doi: 10.1371/journal.pntd.0000788 (PMC2923142; doi:10.1371/journal.pntd.0000788)
Supplement: Table S2 — Full flanking sequences of interation sites of OX3860 into Aedes albopictus. (0.03 MB DOC) [file pntd.0000788.s004.doc]

**Supporting Table S2. Full flanking sequences of integration sites of OX3860 into *Aedes albopictus***.

| **Strain** | **5´ flanking sequence – TTAA insertion site – 3´ flanking sequence** |
| --- | --- |
| OX3860A | ttaatcaactcaacgtacatatgtacagagggatacatcattagtgaaattacgaaagaatccacagctcaggtgagatttgaactcacgacccttttacgctagacaagtgcttttccaactaagctaccgagctaattaatgacaaatgacatggcattttggttggtacaagctaattcaaatctcaccgatc |
| OX3860B | ggccgccagatcttccggatggctcgagtttttcagcaagattcttgaccttgccacagaggactattagaggtaagaataaacattgttggtcaacttcaaagtccacgaggcgtagccgagtctctgcactgaacattgtcagatctcaaagaggtccgacgcgtatgtgccgcaatatatatgacccacttttatgcttcaaactattttttactgatgagataatttcggaaattgtaaaatggacaaatgctgagatatcattgaaacgtcgggaatctatgacaggtgctacatttcgtgacacgaatgaagatgaaatctatgctttctttggtattctggtaatgacagcagtgagaaaagataaccacatgtccacagatgacctctttgatcgacactcctttgtgacttgggtgcggcttgggttgggctgagaacaagatggcgattctaggagttcttttagaggtttcttcaagaattactccagagattctctcaggaatacaccaaatatttcttctggaatccatcaaggggattcttaaggaattctttctgaaattccatcaagatttcattctccaaaaaacctctaatggaagcttcagaaattctaacaagctatccagggctttagcaatttctctcctttggcagacacctgcatacggtgcgcacaagcttagaggtactttaatccaagcagacaaccgaaatgataggtaacattgtcaacgaaagatagatgataacgaacacgagctgacacaaacttcaacgaacgtttagtttaactcgaaattaattaagttaaagataaagaaagttttaacgctaattcttttttgaatatcaggtatatcatattataaggaatacgccttgaagctagatgcgaaattggcc |
| OX3860C | tcgatatttgtatggaaaaatcgccgattattatttaggtattgccatacctgatgtcattattcacgcgttatgcacagaatacacaccagttattattttagatattttacctcttatgcagggctacttaatacctcattcaggttgtaggtattcggttttccatacctgagttagttattcttcagctattttctcctgctcgggtgaggatatccaaaatgcattaaggacaagcttgttggtatccaaagatgggcgccacaatggaaccatgaaaacttaaaccagcgcaccgcaccttcttatgacaaatgagcaagaacaaactaagaattacactattgcctgttgtttacattttaagatttgtgcatttgaacggtgtattgttgaactgggattcaaagagagttgaccttagtttccgaatttctattgagcctgacgtgactagataacccttaaggaatgagtaactcttggtaggatc |
| OX3860D | tcgattttattctctgtcgcattcgctccgaagataatttatttcaaggcacacttaggtgccagccagagtggacgcgtcacgcggcgcggcgcggcgcaatgcggcatttgacaggtcgcgcggcgacgcgcggcagaactccgttcattggaatacagggaaaacaatcatagcatgccagagtgcgcgcggaacgatgcgaagcggaatgcgttttgccgcgcggcgcgcgccagaacagtgcatgcactgtttttgatgcgaaattcgtgcgtttcttgtttgtttacctcggtgattctcaaagttgtaattgaaatggcagaggaaaaaaaagaaattgagcagctaatcgggtccgtatttttacggaaagagttgtgggatcaaacttctcggggataccgaaacagagtgttggtggacaattgttggaaggagcttgccgaagaattcaaagtatctggtgagcatctcattttactaacacaaaattagtattaacgtcattcgttttgcagaagattttttgaagaaaaagtggaagagcctgcgggacaaatatggaaagattttgaggaatcttcccgtatcaaggtccgatgaatatcgtactctctgaattcacccaaaaaatatcttcgaacgccttcagacatgaaatattcatcatctgaatccatttcggcaactttaatgttcaaatacgatgaaacaattggtttgcttttgttttgaacccgcacacttttgcttcgcatcaaataggccttttcagttgacaggtccgtgtatagagataagtgacatttcaacacacgaacactagcgcgaatttttcctcacacaaaaatgcacgccaattcaatggctactcagattgcatatgcaaatattacccaatcga |
| OX3860F | tcgacagacttgtctgaacttcgcaggtacgccatctggaacttccatgtagattgtttcgtttaaacgtccgtgaaatagtatcgccgatgtacaccgactggcaaaacggtcctgatcgtagccagccgagctacctgggcgtaggtcttgtccttgcttttggatgtatcctttcgccacaagccgagttttgtagcgaaccggtctgccgttttcatcttcctttatccaatacacccatttggatttgagaggtttcaccctaagacaggtagccagctgccagacgtcgtttttcttcagcgacattgaccgatagagctgccgttggactccggtttcataggtaggtacataccatggtatacaagtcaacgcagcaccacatccttttcggtagccgagaatccagaagcattgcacgaaccttttcgatgagcgtacaattaaacctctctgccaatccattctgttgtggagaatacgcaaccgtcgctttaatctggaattcttgcgctttgtaccagtttttctgattgttcgagccatactaagtacattgatccaccgtaagttttgaaatattcttaccgaatgctgctgtcgccatcggttcatattcacgaaaccgttcaaaaacttgagacttctttttcatcaagtatattaccgagaaatggctataatcatcaataaacgaaatgaaatagcgggagccatcccacgatagaggatcgattggcc |

Genomic sequences flanking the *piggyBac* insertions of OX3860 lines derived from inverse PCR. The canonical TTAA insertion site is double-underlined. Annealing sites for the primers used to investigate second phase integration into lines OX3860A, B and C are underlined.
